# Supplementary material for: Multi-channel learning for integrating structural hierarchies into context-dependent molecular representation
Source: Nat Commun. 2025 Jan 6;16:413. doi: 10.1038/s41467-024-55082-4 (PMC11704287; doi:10.1038/s41467-024-55082-4)
Supplement: Supplementary file 1 — Supplementary Information [file 41467_2024_55082_MOESM1_ESM.pdf]

Supplementary Information of Multi-channel learning for integrating structural hierarchies into context-dependent molecular representation

Yue Wan[1], Jialu Wu[2], Tingjun Hou[2,*], Chang-Yu Hsieh[2,*], and Xiaowei Jia[1,*]

[1]University of Pittsburgh, Computer Science Department, Pittsburgh, PA 15260, United States
[2]Innovation Institute for Artificial Intelligence in Medicine of Zhejiang University, College of Pharmaceutical Sciences, Zhejiang University, Hangzhou, 310058, China
[*]tingjunhou@zju.edu.cn (T. Hou), kimhsieh@zju.edu.cn (C.-Y. Hsieh), xiaowei@pitt.edu (X. Jia)

1 Representation similarity

We compare the relationship between the conventional fingerprint/descriptor similarity measures and the learned representation distances across channels (i.e., MCD, SCD, and CP), we randomly sample 1000 molecule pairs from ZINC15[1] and examine the correlation between the conventional molecule similarity measures and the learned representation distances. To be more specific, we compute the Tanimoto similarity between molecule ECFP4 fingerprints[2], scaffold ECFP4 fingerprints, and functional groups (i.e., motif) descriptors[3]. We compute and normalize the L2 norm distance between the learned representations. As shown in Figure S1, the representation distance is highly correlated with the conventional similarity measures. This alignment also confirms that the representations learned from different channels indeed capture different global-local perspectives of the molecule.

2 Implicit representation hierarchy

As previously discussed, the representations learned via different channels can elicit different levels of global and local molecule attributes. Molecule distancing focuses on the global view of the molecule, scaffold distancing targets the partial view, and context prediction tackles the local view (i.e., functional groups composition). To better visualize this representation hierarchy, we perform an iterative clustering algorithm over the molecule space of the CHEMBL237 dataset. Specifically, we conduct three stages of clustering with respect to the three channeled representations. Each stage considers the clustered results from the previous stage and further refines the clustered subspace. In hypothesis, representations derived from context prediction (i.e., $\mathbf{h}_g^{[\mathrm{CP}]}$) may result in a coarse-grained clustering in terms of the intra-cluster molecule similarity. This is because molecules with identical functional groups can still elicit structural differences. In comparison, representations from scaffold distancing (i.e., $\mathbf{h}_g^{[\mathrm{SCD}]}$) could yield a less coarse-grained clustering by grouping molecules with similar scaffolds together. Representations from molecule distancing (i.e., $\mathbf{h}_g^{[\mathrm{MCD}]}$) consider structural variations beyond the scaffold, potentially leading to a more fine-grained clustering.

As shown in Figure S2, we first perform clustering with $\mathbf{h}_g^{[\mathrm{CP}]}$. The bar chart illustrates the normalized ratio of intra-cluster motif distance to inter-cluster motif distance from the top-10 largest clusters. It suggests that intra-cluster molecules are more likely to have similar functional groups composition. Peaking inside the clusters, examples show the shared set of functional groups among the grouped molecules, which is denoted as the cluster tag. The two numbers at the x-axis represent the number of unique scaffolds and the cluster size, respectively. It means that the grouped molecules at this stage are not necessarily structurally similar. We further perform the stage two clustering using $\mathbf{h}_g^{[\mathrm{SCP}]}$ on top of the stage 1 results. Again, the bar chart (with darker color) illustrates the normalized ratio of intra-cluster scaffold distance to inter-cluster scaffold distance. Five out of ten clusters contain molecules with same scaffolds. At stage three, the molecule space are further refined using representation $\mathbf{h}_g^{[\mathrm{MCP}]}$. Despite the grouped molecules from stage two already share common scaffolds, the representation could still capture the minor structural differences in the

terminal side chains as shown by the examples. From coarse-grained to fine-grained, the cluster becomes increasingly refined after iteratively applying clustering along the multi-channeled representations. It supports the hypothesis that representations from different channels can capture different views of the molecules from global to local.

3 Roughness comparison

In this section, we report the roughness index (i.e., ROGI)[4] difference between our method and two SSL baseline methods, GraphLoG and MolCLR, prior to fine-tuning on 33 datasets. We also consider the ECFP4 fingerprint[2] as one of the traditional molecule representations used in machine learning. The ROGI value captures the roughness of the property landscape given a representation. Low ROGI value indicates better modellability, and is strongly correlated with the convergence rate and the overall performance at the fine-tune stage. As shown in Figure S4, our representation has better ROGI value compared to other deep molecule representations in 22 out of 33 datasets. Its ROGI value is also lower than that of fingerprint in 18 out of 33 datasets. The advantage of the fingerprint representations is consistent with the finding in[5]. However, it does not guarantee that the fine-tuning performance using fingerprints must outperform other deep learning methods. Roughness metric in general analyzes the overall chemical space, without considering any distribution shift between training and testing sets. It means that representations with smoother structure-property landscape may not necessarily perform better under data splits that introduce distribution shift (e.g., scaffold split in MoleculeNet[6] and stratified split in MoleculeACE[7]). Another reason is because the pre-trained molecule representations serve only as initialization for the embedding space. The model parameters, which are responsible for outputting these representations, also undergo parameter updates during the fine-tuning optimization process. In other words, the updated representations continue to benefit from the flexibility and strength of deep learning models.

4 Convergence rate

We present a comparative analysis of the downstream convergence rates among our proposed method, GraphLoG, and MolCLR in four example datasets (CHEMBL237_Ki, CHEMBL262_Ki, CHEMBL2034_Ki, and CHEMBL234_Ki). As illustrated in the Figure S5 , our representation facilitates faster convergence rate in terms of both the training and validation R-squared value. It suggests that our representation exhibits better downstream transferability, which is consistent with the conclusions on modellability discussed in Section 3 in the main text. To further understand the efficacy of our composite representation achieved through prompt weight selection, we also consider the downstream performance when solely fine-tuned from individual channels, represented by Ours_MCD, Ours_SCD, and Ours_CP. A notable observation is that their performance often exhibits high volatility, such that both the training and validation R-squared value oscillates significantly between epochs. One plausible explanation is that each corresponded prompt-guided aggregation module considers atoms with varying degrees of importance. In other words, individual aggregations may only tackle a partial view of the molecule, especially for the SCD and CP channels. Given that the aggregation modules remain fixed during fine-tuning, these partial perspectives may fall short in comprehensively learning the structure-property relationship.

5 Representation robustness

In the main text, we evaluate the shift in representation space with three methods (Ours_{GIN}, GraphLoG[8], and MolCLR[9]) when fine-tuned on the CHEMBL237_Ki dataset. In this section, we further analyze the representation performance under the few-shot scenarios. Specifically, we evaluate the fine-tuned results of the validation set using only 1%, 5%, 10%, 50%, and 100% of the training data, corresponding to the 1-shot, 5-shot, 10-shot, 50-shot, 100-shot columns as shown in Figure S6. Each visualization shows what the representation space looks like after training for 100 epochs. Following the work in[7], instead of randomly sampling the training data, we perform stratified sampling by first applying k-means clustering using the molecule fingerprint, and selecting the required amount of data samples that are diverse across the clustering. We report the same Rand index[10] and cliff-noncliff distance ratio as the analysis in the main text. As shown in the figure, even with 1% of training data, the representation space of MolCLR distorts significantly. The small rand index implies that large amount of structural information is lost, which can also be interpreted as label overfitting. GraphLoG, compared to MolCLR, shows a more balanced representation space mapping throughout all few-shot scenarios, also indicated by its consistently stable rand index around 0.24. Our method also maintains high Rand index across all few-shot scenarios. When comparing the cliff-noncliff distance ratio, our method consistently performs better than others with larger distance ratio. As shown by the validation R-squared curves, our method also achieves better performance in terms of the highest R-squared value, faster convergence rate, and training stability.

6 Case study in prompt-guided node aggregation

To interpret the prompt-guided aggregation, we randomly choose two out-of-distribution molecules from BBBP[6] (beyond the pre-train datasets) and visualize their node attention scores, as shown in Figure S7 and Figure S8. The highlighted scaffold corresponds to the Bemis-Murcko scaffold, while different functional groups are highlighted using different colors. For illustrative purposes, not all the functional groups are shown because of the atom overlaps. The node attention id corresponds to #<channel_id>.<head_id>. For example, node attention #1.1 is taken from the first attention head in the first channel (MCD). For the predicted node attention, darker color indicates higher atom contribution, and vice versa. As illustrated by the figures, the node aggregation module is able to capture the expected atom importance. All atoms contribute to the molecular representation of the MCD channel, while only the atoms belonging to the scaffold are treated important for the SCD channel. When it comes to the node attentions computed for the CP channel, the attention scores of each head is a lot messier than before. One of the reasons is because CP is not only doing functional group composition prediction, but also masked subgraph prediction. Also, it is impossible to have a one-to-one mapping from attention heads to functional groups. However, we could still observe some interesting local patterns captured by the attention. For example, the node attention #3.4 in Figure S8 seems to capture the bicyclic compound, the carbonyl group, as well as the thioether of the molecule.

Even though the prompt-guided node aggregations are fixed during fine-tuning, the graph encoder (e.g, GNN) is still tunable. Therefore, we further investigate whether the aggregation patterns hold during fine-tuning, especially for the first two channels. Surprisingly, we realize that there is only a small shift in aggregation patterns. As shown in Figure S9, aggregation patterns from the first two channels are visualized at epoch 20, 50, and 100 when fine-tuning the BBBP dataset. During fine-tuning, the score distribution of attention #1.1 changes slightly, but it continues to span across all atoms. For attention #2.1, the non-scaffold atoms, especially for the carbon chain in the example, begin to receive increased attention. However, their contributions to the aggregation remain lower than those of the scaffold atoms. We hypothesize that the fixed aggregation module acts as a constraint, limiting the degree of freedom of the graph encoder module during fine-tuning to ensure that the aggregation output is meaningful.

7 Ablation study

To gain a clearer understanding of the effects of each component on downstream performance, we conduct a comprehensive ablation study. Specifically, the ablation controls the main components in our pre-training design (i.e., adaptive margin loss, multi-channel learning framework, and regularizations) and fine-tuning strategies (i.e., simple concatenation of channel-wise representations versus learnable prompt selection module). Additionally, we also examine the relationship between individual channels and the chemical knowledge necessary for learning molecular properties.

7.1 Pre-train components

We compare the model performance on the MoleculeACE benchmark across 6 different pre-training settings: 1. The full pre-training setting, corresponding to the best model configuration described in the main text. 2. Without any regularizations. 3. Without intra-channel regularization on node aggregation patterns. 4. Without inter-channel regularization on channel alignment. 5. Replacing the adaptive margin loss with the conventional margin loss. 6. Replacing the multi-channel learning with conventional multi-task learning, where there is only a single aggregation channel that learns all the pre-trained tasks. All settings, including the full setting, are pre-trained using GIN[11] as the model backbone for 40 epochs. Figure S10 presents the performance comparison across the 30 MoleculeACE datasets, measured by the average test R-squared value. As before, performance on each dataset is averaged over three independent runs using stratified splits. The error bars represent the average standard deviation across the three runs for the 30 datasets. As shown in the plot, the full setting achieves the highest performance, with an average R-squared value of 0.6288. The removal of regularizations and the replacement of the adaptive margin loss both result in a slight drop in performance. Notably, intra-channel regularization in node aggregation patterns appears to play a more important role in pre-training compared to inter-channel alignment regularization. The largest performance drop is observed in the multi-task learning setting. It is important to note that the only difference between this setting and the multi-channel learning setting is that the latter framework learns the same set of tasks in separate channels. This highlights the effectiveness of multi-channel learning, as it decomposes the pre-trained tasks based on different aspects of chemical knowledge, allowing for the combination of pre-trained knowledge in a task-specific manner. The full performance table is shown in Table S4.

7.2 Fine-tune strategies

Additionally, we examine three different strategies for leveraging the learned channel-wise representations during fine-tuning:

1. Simple concatenation (Concat) of the channel-wise representations without applying any channel weights. The prediction is made using a task-specific fully connected layer (FC) as the prediction head on top of the concatenated representations.

$$\text{prediction} = \text{FC}([h_g^{[\text{MCD}]}, h_g^{[\text{SCD}]}, h_g^{[\text{CP}]}]) \tag{1}$$

2. Aggregation of channel-wise representations via a learnable prompt tuning module $\tau_\theta(\cdot)$, which corresponds to the primary setup in our experiments. In this case, τ_θ is a tunable prompt weight (PW) vector consisting of three elements, each representing the logit l for a specific channel. Its initialization is guided by the ROGI value[4]. Additionally, a temperature t is used to control the sparsity of the channel selection. A smaller temperature value results in a sharper distribution, concentrating the probability mass on fewer channels. In this ablation, we compare the effect of different temperature values $t \in \{1, 0.7, 0.3\}$. The best performance reported in the main text is achieved using $t = 0.7$. It is important to note that this method is task-specific but sample-agnostic.

$$\tau_\theta = [l_0, l_1, l_2],\ \alpha = \text{Softmax}(\tau_\theta / t),\ \text{prediction} = \text{FC}(\sum_i \alpha_i h_g^i) \tag{2}$$

3. Aggregation of channel-wise representations using a Mixture-of-Experts[12] (MoE) alike paradigm. In this approach, $\tau_\theta(\cdot)$ is also learned to aggregate the channel-wise representations, but in a different manner. Specifically, for each molecular graph, a gating network (e.g., a fully connected layer) takes the graph representation obtained via mean pooling as input and outputs a set of importance weights for each expert (i.e., channel). Similar to the previous method, the composed representation is a weighted sum of the channel-wise representations. Again, different temperature values t are explored. Notably, this is both a task-specific and sample-specific aggregation method, meaning that channel importance can vary between different molecules. While this approach enhances the model's expressiveness, it also reduces interpretability and increases the risk of overfitting by the additional complexity.

$$\tau_\theta = \text{GATE}(h_g^{mean}),\ \alpha = \text{Softmax}(\tau_\theta / t),\ \text{prediction} = \text{FC}(\sum_i \alpha_i h_g^i) \tag{3}$$

This experiment is conducted using the best model checkpoint of GIN from the main experiments. The average test R-squared value across the 30 datasets in MoleculeACE was measured, with performance on each dataset averaged over three independent runs using stratified splits. The error bars represent the average standard deviation across the three runs for the 30 datasets. As shown in Figure S11, Sparse PW with $t = 0.7$ achieves the best performance. The Concat method, which evenly utilizes all available information, performs slightly worse than PW. This highlights the advantages of selectively utilizing pre-trained information rather than using everything indiscriminately. Surprisingly, the MoE approach performs the worst, despite its ability to select channels via τ_θ. One of the reasons could be its prone to overfitting due to the additional complexity. Additionally, unlike the learnable PW, MoE does not incorporate prior information from the ROGI measure, highlighting the advantages of ROGI-guided initialization. Moreover, it is unsure whether the graph representation derived from mean pooling is suitable for inferring the importance of each channel. It would be interesting to further explore effective aggregation methods for channel-wise representations in future work. Another interesting observation is the tradeoff between information sparsity and completeness. For both PW and MoE, the performance improves from $t = 1$ to $t = 0.7$ but drops from $t = 0.7$ to $t = 0.3$. This suggests that some degree of channel selectivity is beneficial. However, as the temperature continues to decrease, the model will tend to rely on a single channel for predictions, ignoring information of other channels. This approach proves to be less effective than leveraging multiple channels. Detailed performance is included in Table S5.

8 Interpretability of channel selectivity

Since our method can build task-specific, context-dependent representations through channel aggregation, it would be interesting to see how representation from each channel affects the model performance, and whether this aligns with the chemical knowledge required for the downstream property prediction. This analysis mainly tackles the interpretability of the learnable prompt modules during fine-tuning.

8.1 Case study in channel activation

Recall that each channel focuses on a specific aspect of the molecule: channel MCD focuses on molecular similarity, channel SCD focuses on scaffold similarity, and channel CP focuses on local patterns like functional group composition. To keep the experiment simple, we focus solely on whether a channel is activated, disregarding the exact weight of each channel. Consequently, we evenly assign the prompt weights across the activated channels. For example, if only MCD and SCD are activated, the prompt weights would be [0.5, 0.5, 0]. As shown in Table S1, we choose four binding potency prediction datasets, and run Our_{GPS} model on multiple settings with different channel activation. In order to quantify which part of the chemical knowledge is more beneficial for solving the tasks, we decide to analyze the quantitative structure-property relationship (QSPR) between the potency label and the structural features of molecule fingerprint, scaffold fingerprint, and the binary functional group descriptors. We formulate our QSPR metric as the pearson correlation between the fingerprint/descriptor differences (i.e., $1-$ Tanimoto similarity) and the potency label differences. ECFP4 fingerprint is used. We then normalize the three correlation values. Since this QSPR metric (also true for most QSPR metrics) does not account for distribution shift, it can barely correlate with the model performance comparison under the original stratified sampling data splits[7]. Therefore, the performance in the table corresponds to the average R-squared value across three runs under the random split. By examining the columns where only one channel is activated, the performance shows a high correlation with the QSPR metric. For example, in the CHEMBL236_Ki dataset, the functional group descriptor has the lowest normalized correlation value of 0.203 with the potency labels. Correspondingly, the R-squared value is lowest when using the CP channel alone. However, when multiple channels are activated, the relationship between channel activation and QSPR metric becomes less correlated. It is not necessarily true that two channels with high corresponding QSPR values will lead to better performance. A typical example is when activating channels MCD and SCD on the CHEMBL237_Ki dataset. Even though the molecule and scaffold fingerprints show high QSPR values, using both channels worsens the performance. This could be explained by feature redundancy within the two channels that degrades the performance. It also explains why activating all channels simultaneously may not lead to the best performance, which is consistent with the results in Figure S11.

8.2 Relationship between optimized prompt weights and QSPR metrics

We further examine the relationship between the optimized prompt weights and the QSPR metrics, which approximate the chemical knowledge required for solving the tasks. The key difference between this analysis and the previous case study is that the optimized prompt weights are fully machine-learned rather than handcrafted. They result from both the ROGI-guided initialization and subsequent optimization. To begin, we retrieve the optimized prompt weights (PW) from the best validation model after fine-tuning on each of the 30 datasets in MoleculeACE using the random split. We then collect the same QSPR measures from above (i.e., the normalized correlation between representation difference and label difference) for all datasets. Finally, we conduct a principal component analysis (PCA) on these two sets of normalized vectors and visualize their relationship in a scatter plot using the computed first components.

As shown in Figure S12, we observe some correlations between the optimized prompt weights (PW) and QSPR measures. However, the alignment is far from perfect. One possible reason could be the feature redundancy hidden within the channel-wise representations, which may influence the model's decision. Additionally, due to the black-box nature of machine learning models, the problem may be solved in ways that differ from human interpretation. This suggests that hidden information might be processed differently by the model, which requires further investigation. Moreover, it is important to note that large-scale pre-training does not necessarily endow the model with perfect capability in solving the pre-train tasks. Learning multiple tasks simultaneously can still pose challenges, even within the multi-channel learning framework. This indicates that there may be information gaps between the learned channel-wise representations and the actual chemical knowledge they are meant to capture. An intriguing direction for future work would be to analyze how the model's actual capabilities, as learned during pre-training, influence fine-tuning performance.

9 Framework extension with other input representations

As mentioned in the Discussion section of the main text, it would be interesting to apply our framework to input representations other than 2D molecular graphs. However, depending on the downstream application, it is uncertain whether one input representation would outperform the others in terms of model performance. Here, we provide a brief discussion on the possible ways of extending our framework for reference purposes. Two scenarios are discussed: (1) changing the single-modal representation with/without altering the pre-training tasks, and (2) utilizing multi-modal representations along with cross-modal pre-training tasks.

Regarding the single-modal setting (i.e., using only SMILES or 3D graphs), our framework can be easily adapted to both scenarios. The primary adjustment involves changing the encoder accordingly (e.g., a Transformer-based encoder for SMILES and a 3D-GNN for 3D geometry). For the pre-training tasks, special attention must be paid to the definition of subgraph under different modalities. In the case of a SMILES string, its subsequences do not necessarily correspond to valid substructures within the molecule. We would need to extract the subgraph from molecular graph and map it back to the SMILES sequence. For 3D geometry, graphs are often defined differently (e.g., neighbors are defined by geometric distance rather than chemical bonds[13]). Hence, we need to ensure that graph operations are consistent with the given definition. Scaffold-invariant perturbations will not be affected by the change in input representations. Essentially, new molecules are created by the perturbation. On the other hand, there are a lot more self-supervised learning tasks that can be performed with 3D geometry (e.g., 3D coordinates recovery[14] and bond angle prediction[15]), which can be incorporated into additional learning channels. However, we believe it is crucial to group these tasks into distinct categories based on commonalities, such as the global-local perspective of the molecule. As suggested in Table S1, the fine-tuning performance could be affected by the feature redundancy across channels.

One potential way to extend the framework with multi-modal representations is to leverage and group the pre-training tasks into distinct channels by information level (e.g., 1D, 2D, or 3D tasks). Additionally, we could incorporate cross-modal self-supervised learning tasks that explicitly guide channel alignment (e.g., contrastive learning between 2D topological graphs and 3D geometry[16]). One potential advantage is that communication between different encoders may enhance the learning of individual encoders. Moreover, the framework may become more transferable to tasks that require different levels of knowledge (e.g., structure composition versus conformation information).

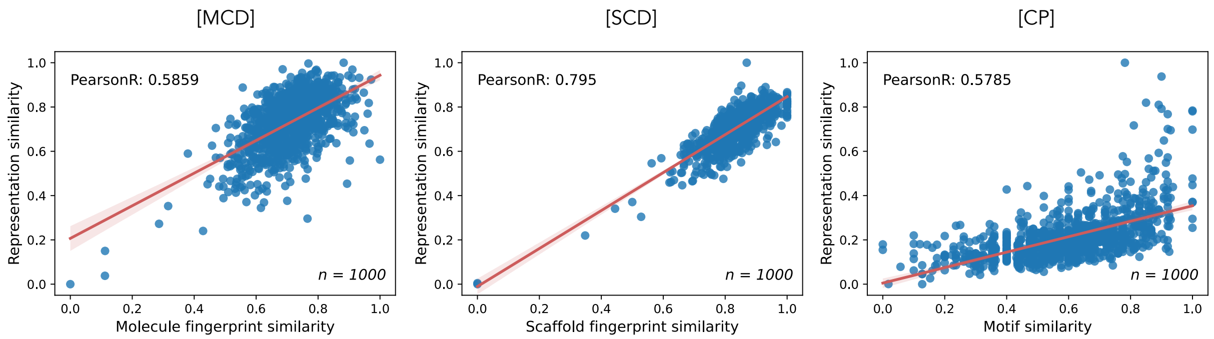

Figure S1. Comparison of conventional similarity measures and representation distance. For illustration purposes, 1000 molecule pairs are randomly sampled from ZINC15[1]. The x-axis represents the molecular similarity measured by Tanimoto similarity over structural features (i.e., fingerprint, scaffold fingerprint, functional group composition vector), while the y-axis represents the similarity measured by the normalized L2 norm over learned representations. Pearson correlation coefficient is reported.

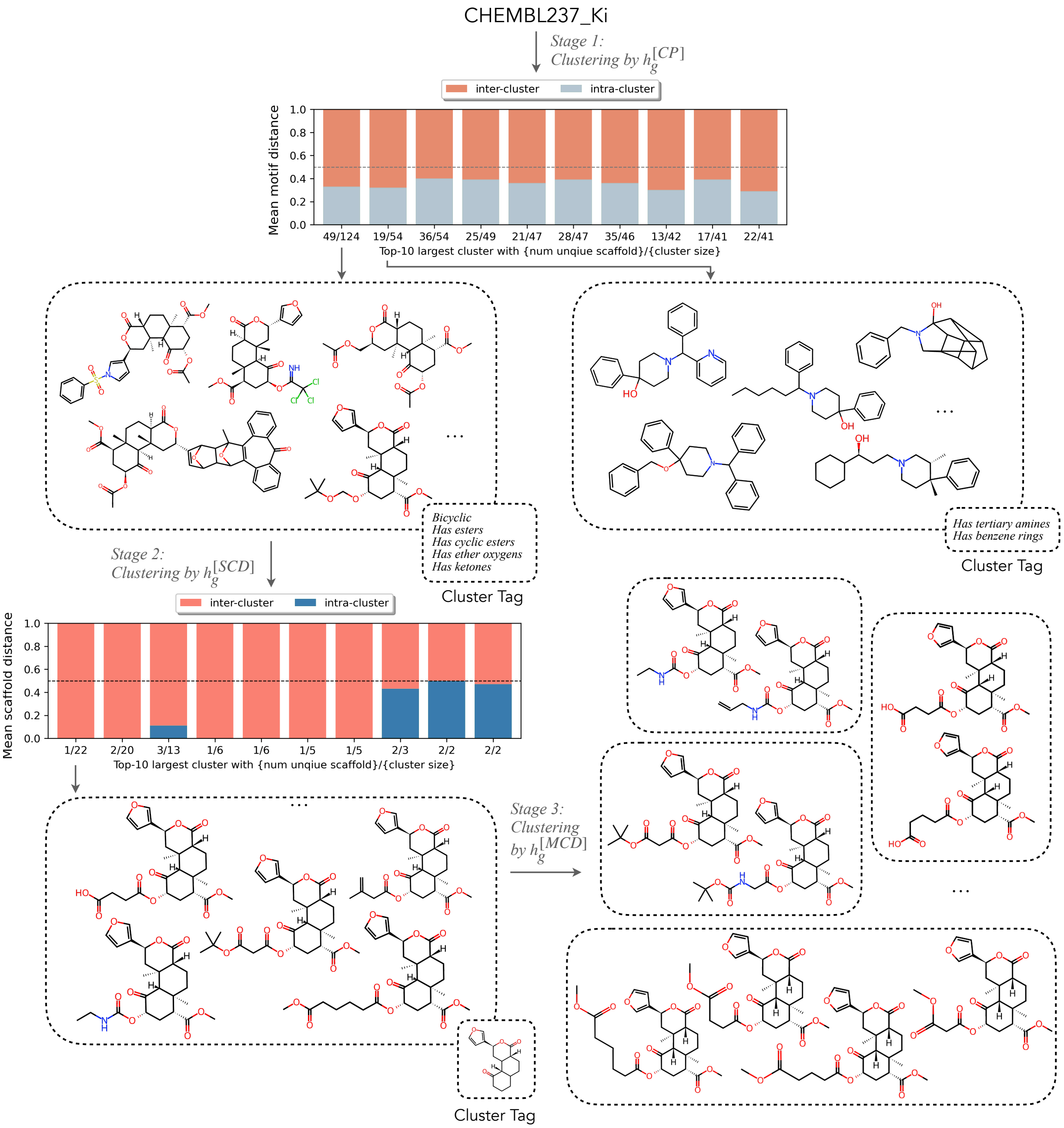

Figure S2. Hierarchical clustering by the multi-channel representation. The molecules in CHEMBL237_Ki[7] ($n = 2602$) are initially clustered using the learned representation $\mathbf{h}_g^{[CP]}$, which emphasizes the local composition of each molecule. The bar chart shows the inter-cluster and intra-cluster difference of average motif distance, measured by 1 - Tanimoto similarity over the functional group composition vectors. Each cluster is then further divided into subsequent sub-clusters using $\mathbf{h}_g^{[SCD]}$ and $\mathbf{h}_g^{[MCD]}$. Scaffold distance is measured by the 1 - Tanimoto similarity over the scaffold fingerprint. The properties of each sub-cluster are indicated by their cluster tags and visualized through sample illustrations.

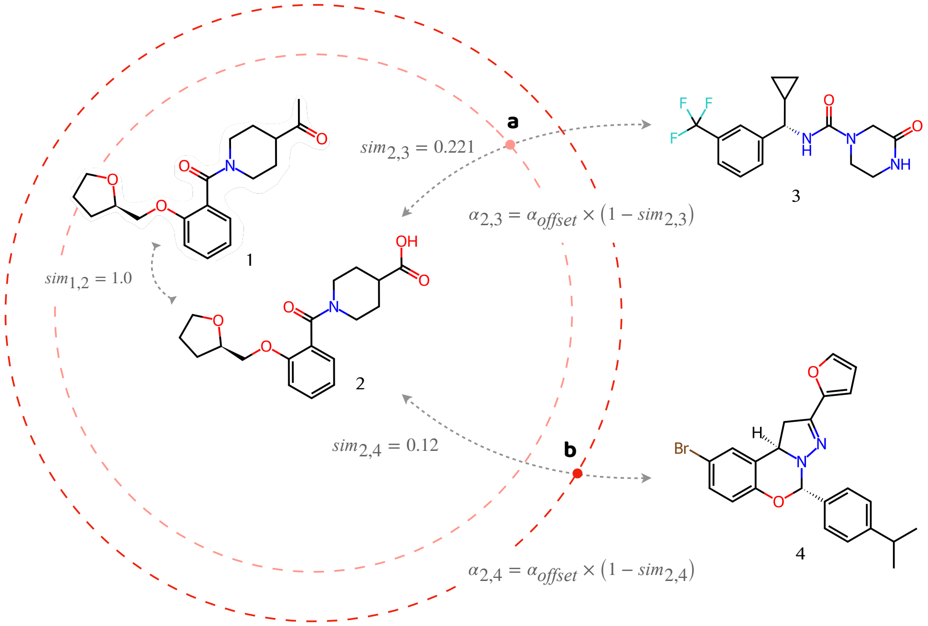

Figure S3. Adaptive margin loss. Triplet examples *<1,2,3>* and *<1,2,4>* considered in scaffold distancing adaptive margin loss. $sim_{i,j}$ represents the scaffold fingerprint Tanimoto similarity between compound i and j, while $\alpha_{i,j}$ corresponds to the computed margin (indicated by the dashed line). Note that, in this example, (a) the margin $\alpha_{2,3}$ is smaller than (b) the margin $\alpha_{2,4}$ because of the similarity difference. Both compound 3 and 4 lie outside of the margin, hence making the triplet loss $\ell_{1,2,3} = \ell_{1,2,4} = 0$. However, the actual representation distance between *<2, 3>* and *<2, 4>* may not necessarily correlate with the computed margins, as shown by the figure. This motivates our quadruplets formulation.

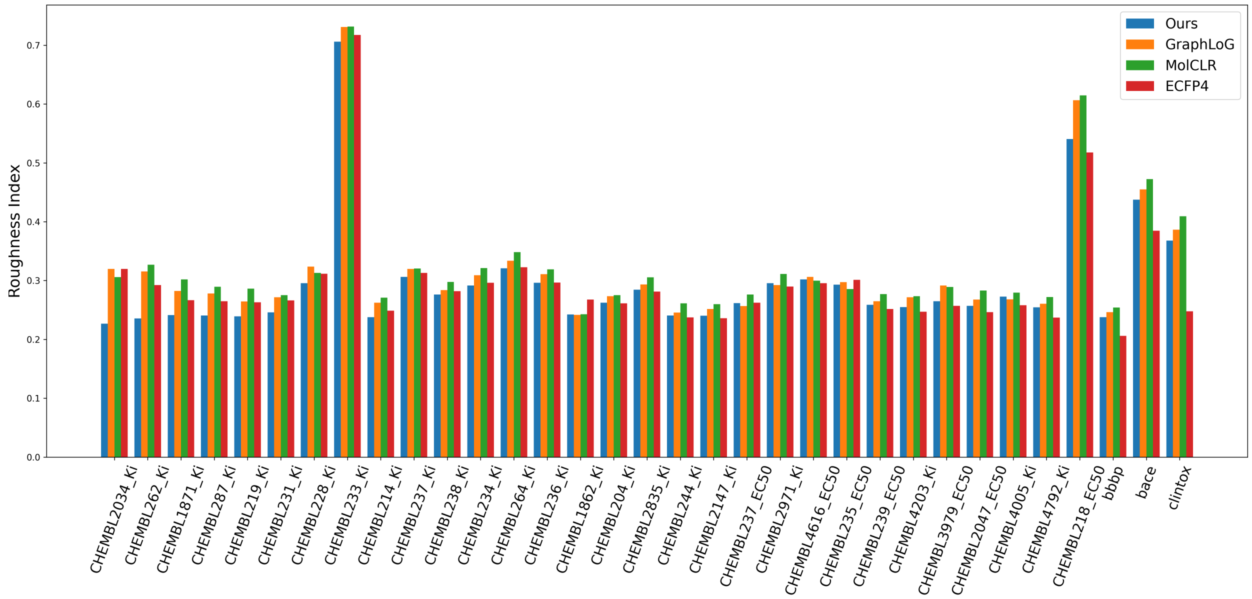

Figure S4. **Roughness index (ROGI) comparison.** The computed ROGI[4] value of the representations (Ours$_{\text{GIN}}$, GraphLoG[8], MolCLR[9], and ECFP4 fingerprint[2]) against molecular property measures in 33 datasets[6,7]. Lower ROGI value indicates smoother molecular property landscape. The bars are arranged from left to right based on the descending order of the ROGI value differences between our representation and the most effective alternative representation.

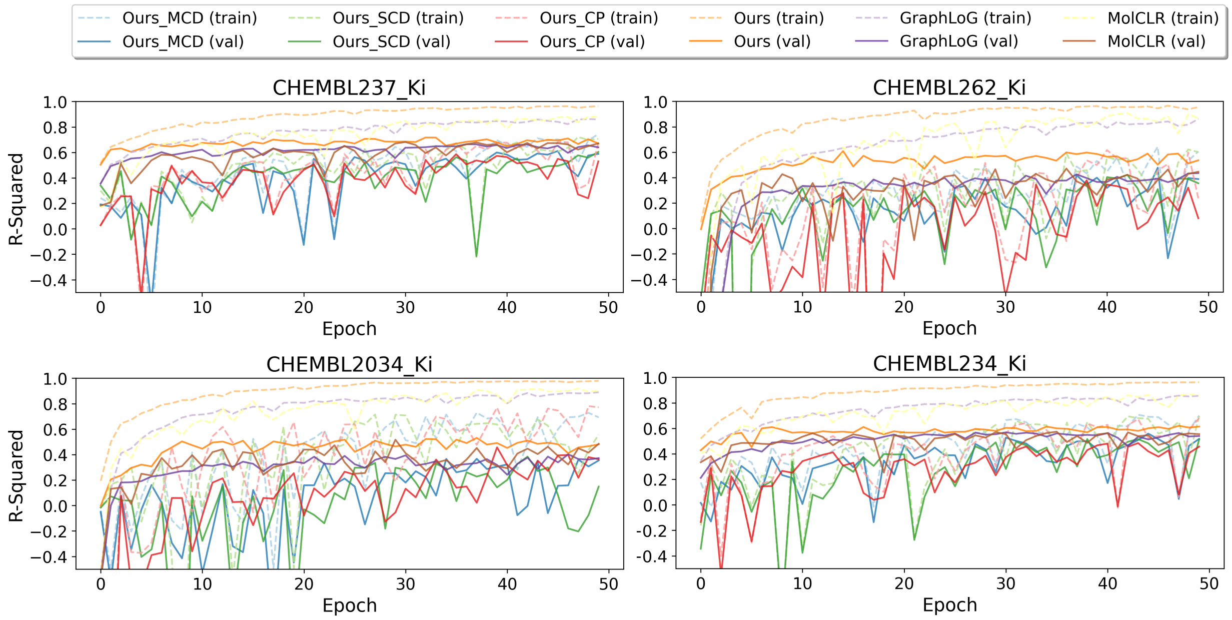

Figure S5. Convergence rate at fine-tuning. R-squared curves of Ours$_{\text{GIN}}$, GraphLoG[8], MolCLR[9] on both training and validation set of four datasets are ploted. Ours_MCD, Ours_SCD, and Ours_CP represent the use of only the corresponding channel for fine-tuning. The dashed line represents the training R-squared value, while the solid line corresponds to the validation R-squared value.

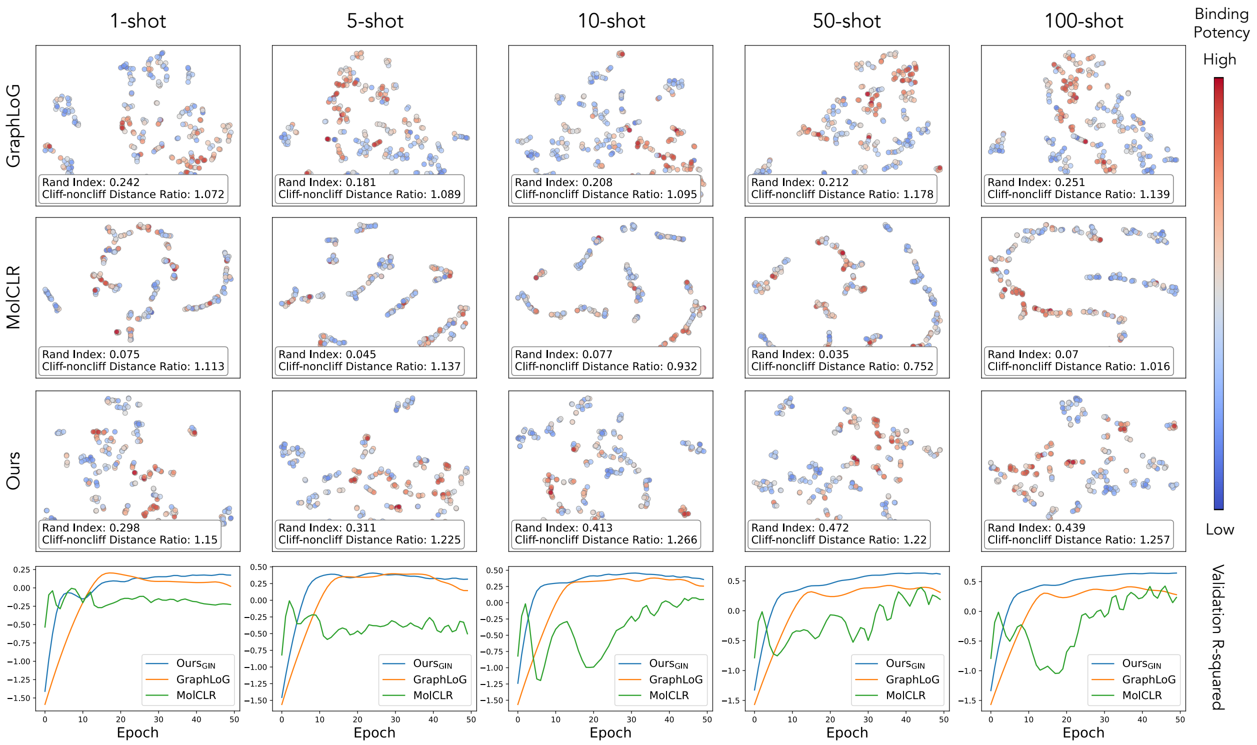

Figure S6. Representation space probing (few-shot scenario). The dynamics of molecule representation space of Ours_{GIN}, GraphLoG[8], and MolCLR[9] under five few-shot settings on dataset CHEMBL237_Ki[7] ($n = 2602$). For each row, a 2D view of the representation space of the validation set is visualized. The dot coloring represents the normalized potency labels. Rand index[10] and cliff-noncliff distance ratio are reported, along with the validation R-squared value along the training process (bottom row).

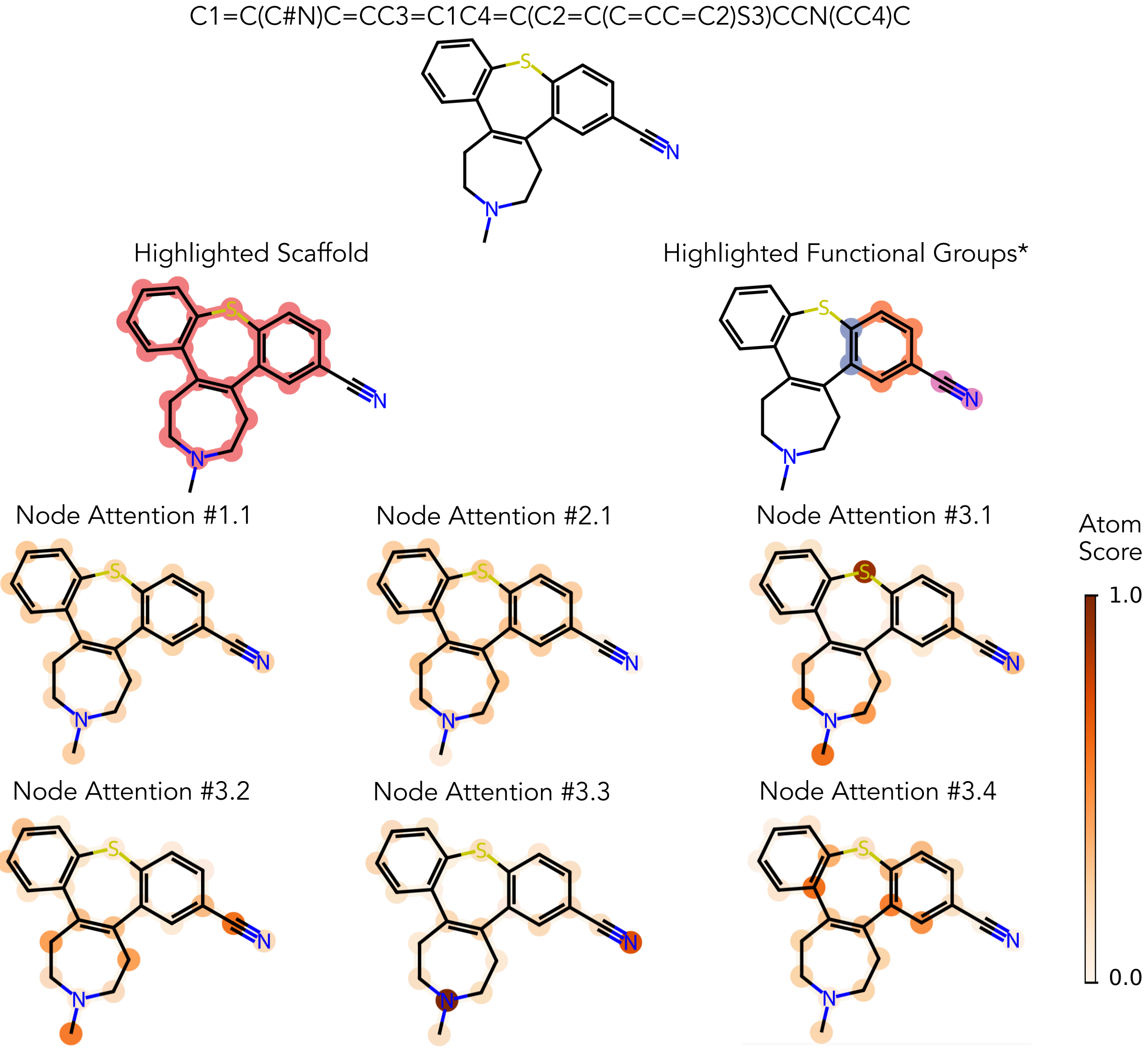

Figure S7. Case study of prompt-guided aggregation. Visualization of atom aggregation attention on molecule 18-methyl-8-thia-18-azatetracyclo[13.5.0.02,7.09,14]icosa-1(15),2(7),3,5,9,11,13-heptaene-4-carbonitrile, along with its scaffold and functional groups. For illustration purposes, only certain functional groups are highlighted considering the atom overlaps (indicated by the *). The node attention id follows the format of #<channel_id>.<head_id>. Six different attention distributions are shown from the prompt-guided aggregation. Darker color means higher atom importance.

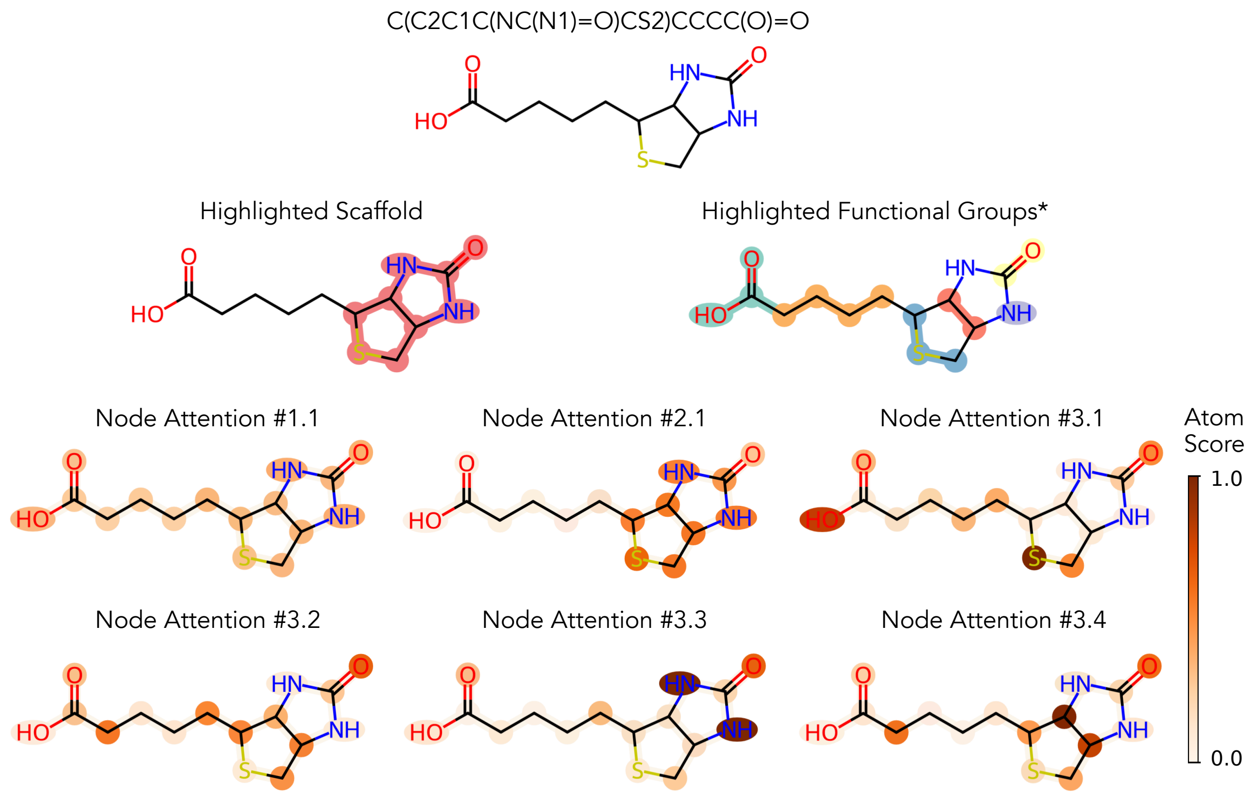

Figure S8. Case study of prompt-guided aggregation. Visualization of atom aggregation attention on molecule 5-(2-oxo-1,3,3a,4,6,6a-hexahydrothieno[3,4-d]imidazol-4-yl)pentanoic acid, along with its scaffold and functional groups. For illustration purposes, only certain functional groups are highlighted considering the atom overlaps (indicated by the *). The node attention id follows the format of #<channel_id>.<head_id>. Six different attention distributions are shown from the prompt-guided aggregation. Darker color means higher atom importance.

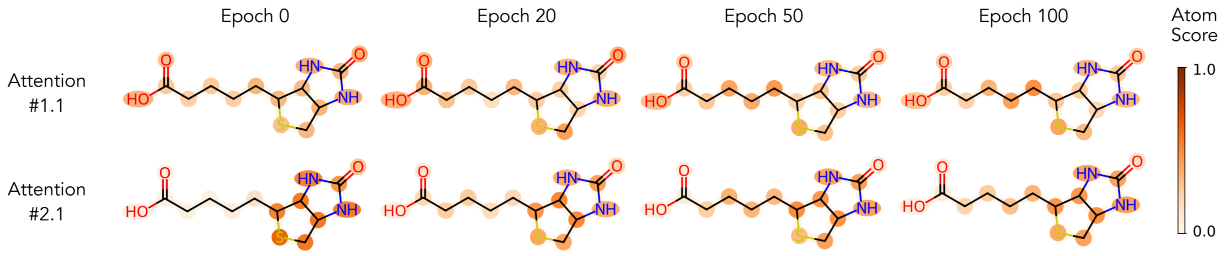

Figure S9. Case study of prompt-guided aggregation during fine-tuning. Visualization of aggregation attention on molecule 5-(2-oxo-1,3,3a,4,6,6a-hexahydrothieno[3,4-d]imidazol-4-yl)pentanoic acid, when fine-tuning BBBP[6] at epoch 20, 50, and 100. The node attention id follows the format of #<channel_id>.<head_id>. Darker color means higher atom importance.

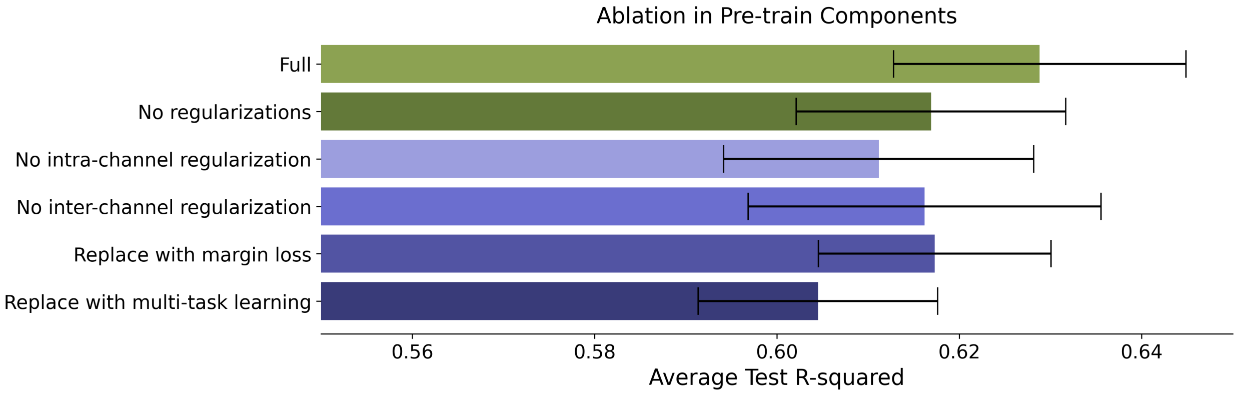

Figure S10. Ablation study in pre-train components. The average performance on the 30 datasets in MoleculeACE[7] under 6 pre-train settings. The error bars represent the average standard deviation of the standard deviation across the three independent runs for each dataset. See Table S4 for more details.

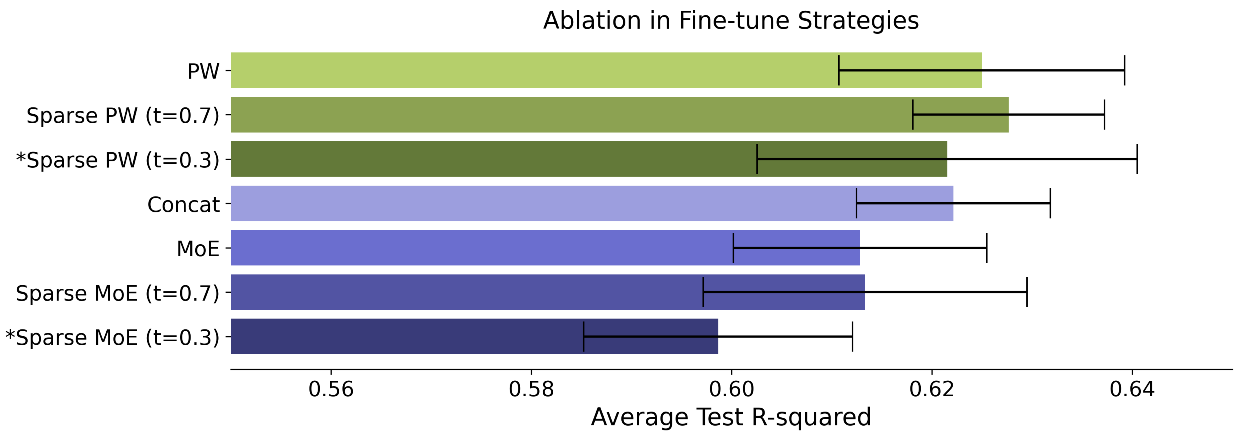

Figure S11. Ablation study in fine-tune strategies. The average performance on the 30 datasets in MoleculeACE[7] using 7 fine-tune strategies of leveraging the channel-wise representations. The error bars represent the average standard deviation of the standard deviation across the three independent runs for each dataset. See Table S5 for more details.

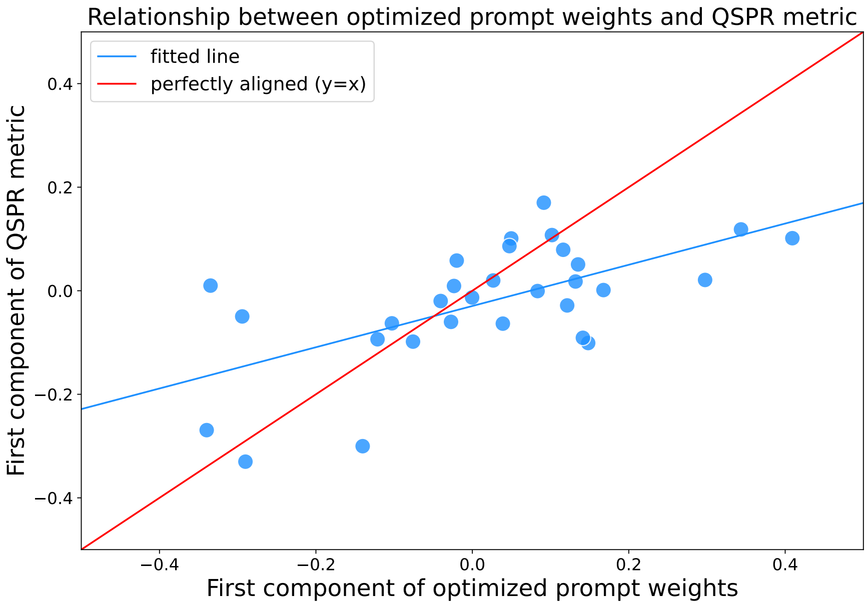

Figure S12. **Relationship between optimized prompt weights (PW) and QSPR measure.** The optimized prompt weights is collected from the best validation model during fine-tuning. This experiment is done using random splits on the 30 datasets ($n = 30$) in MoleculeACE[7]. QSPR stands for the quantitative structure-property relationship measures we used to approximate the required chemical knowledge for solving the tasks. Principle component analysis (PCA) is performed and the relationship between the first components of PW and QSPR are visualized, as well as the fitted line.

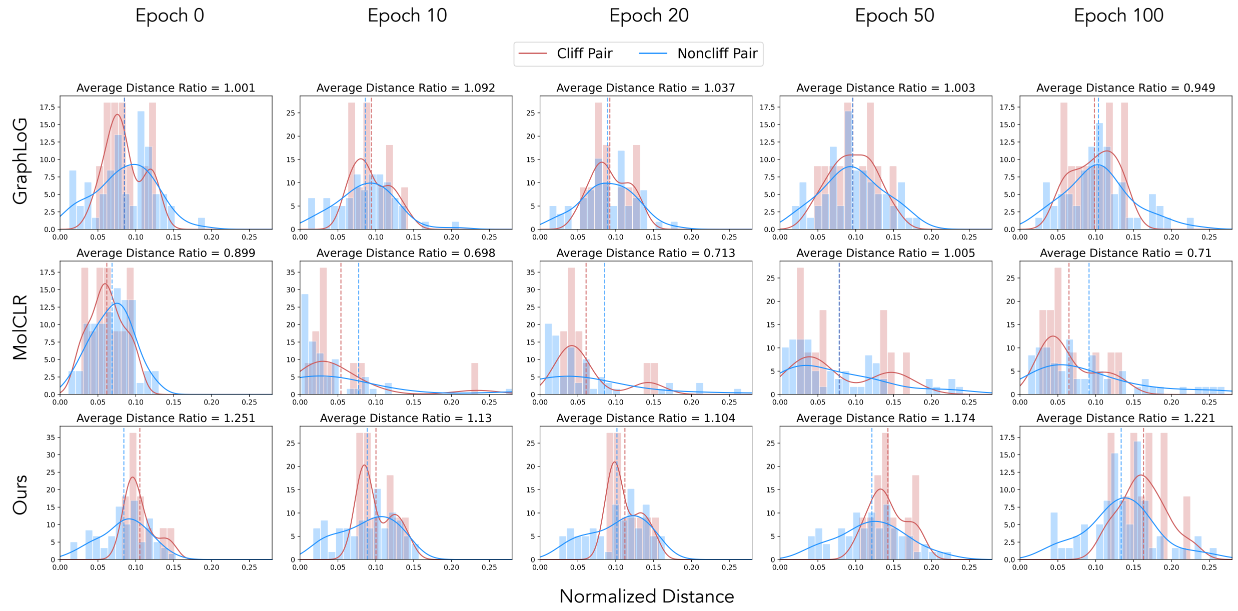

Figure S13. Histogram of distances of cliff and non-cliff pairs. The shift in cliff distances and non-cliff distances during the representation space probing in Figure 3. For both GraphLoG[8] and our method, non-cliff and cliff matched molecule pairs (MMPs) are pushed further apart along the training process. This is understandable, as the learned representations become more label-oriented rather than structure-oriented during fine-tuning, leading to some loss of structural information. However, it remains important for the model to maintain distance differences for understanding of activity cliffs throughout fine-tuning, which is particularly true for our method. In contrast, MolCLR[9] exhibits more oscillatory behavior, with less consistent distance distribution patterns during fine-tuning. This corresponds to the drastic shifts of MolCLR's representation space shown in the main text.

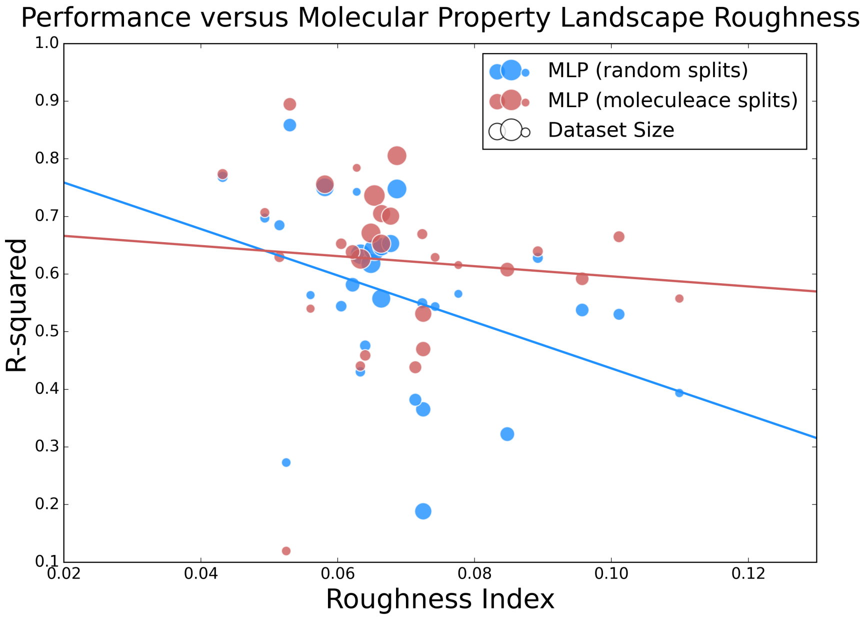

Figure S14. Relationship between ROGI and model performance under different splits. The test performance of MLP with ECFP4 fingerprint on the 30 datasets ($n = 30$) in MoleculeACE[7], averaged across three independent runs, in relation to the roughness of molecular property landscapes[4] under the random splits and the MoleculeACE's stratified splits. The size of the dots represents the dataset size, and the slope of the fitted line indicates the correlation. It demonstrates how the correlation between measures of chemical space discontinuity and the model performance gets affected by the designated data splits. This explains our choice of random splits for all experiments involving QSPR analysis.

Table S1. Channel activation ablation. The performance comparison on CHEMBL236_Ki, CHEMBL1871_Ki, CHEMBL228_Ki, and CHEMBL237_Ki datasets[7] when enabling different channels. QSPR stands for the quantitative structure-property relationship metric we used. We manually assign the prompt weights to the channels regarding their activation. This experiment is done using random splits.

Dataset	QSPR	Channel	Activation						
CHEMBL236_Ki	0.423	MCD	✓			✓		✓	✓
	0.374	SCD		✓		✓	✓		✓
	0.203	CP			✓		✓	✓	✓
	Performance		0.765	0.758	0.756	0.771	0.764	0.776	0.772
CHEMBL1871_Ki	0.391	MCD	✓			✓		✓	✓
	0.272	SCD		✓		✓	✓		✓
	0.338	CP			✓		✓	✓	✓
	Performance		0.523	0.469	0.541	0.529	0.515	0.526	0.518
CHEMBL228_Ki	0.402	MCD	✓			✓		✓	✓
	0.266	SCD		✓		✓	✓		✓
	0.332	CP			✓		✓	✓	✓
	Performance		0.667	0.644	0.662	0.644	0.667	0.667	0.662
CHEMBL237_Ki	0.461	MCD	✓			✓		✓	✓
	0.501	SCD		✓		✓	✓		✓
	0.039	CP			✓		✓	✓	✓
	Performance		0.769	0.772	0.762	0.749	0.765	0.756	0.757

Table S2. Fine-tuning performance (R-squared) of 12 methods on 30 binding potency prediction tasks in MoleculeACE[7] using stratified splits. $C2047_{EC}$ is short for the CHEMBL2047_EC50 dataset, while GraLoG=GraphLoG, GROV=GROVER, and MoLF=MoLFormer. The performance is averaged across three independent runs, where standard deviation is also shown. Zero standard deviation indicates that the value is below 0.01. Best performance is shown in bold.

Dataset	MLP	GIN[11]	GraLoG[8]	MolCLR[9]	GROV[3]	MoLF[17]	GEM[15]	UniMol[14]	KANO[18]	KPGT[19]	$Ours_{GIN}$	$Ours_{GPS}$
$C1862_{Ki}$	0.71±0.01	0.71±0.03	0.69±0.03	0.76±0.03	0.75±0.01	0.71±0.0	0.79±0.01	0.81±0.01	0.76±0.01	**0.82±0.01**	0.77±0.01	**0.82±0.01**
$C1871_{Ki}$	0.54±0.02	0.42±0.05	0.33±0.03	0.41±0.09	0.42±0.06	0.47±0.0	0.46±0.04	0.49±0.06	0.55±0.02	**0.63±0.01**	0.54±0.01	0.59±0.01
$C2034_{Ki}$	**0.63±0.01**	0.3±0.06	0.56±0.03	0.4±0.1	0.52±0.02	0.6±0.02	0.44±0.04	0.34±0.04	0.5±0.04	0.55±0.03	0.52±0.02	0.55±0.04
$C2047_{EC}$	**0.62±0.01**	0.41±0.08	0.52±0.02	0.44±0.09	0.59±0.02	0.6±0.02	0.38±0.02	0.34±0.08	0.25±0.11	0.25±0.02	0.32±0.05	0.31±0.02
$C204_{Ki}$	0.76±0.01	0.76±0.02	0.71±0.02	0.72±0.02	0.66±0.01	0.73±0.0	0.78±0.01	0.81±0.01	0.77±0.01	0.82±0.01	0.79±0.01	**0.83±0.0**
$C2147_{Ki}$	0.89±0.0	0.88±0.01	0.82±0.01	0.87±0.02	0.85±0.01	0.86±0.01	0.85±0.01	0.87±0.01	0.86±0.01	**0.9±0.0**	0.87±0.0	0.89±0.0
$C214_{Ki}$	0.63±0.0	0.63±0.01	0.58±0.02	0.6±0.02	0.45±0.09	0.59±0.02	0.6±0.03	0.66±0.01	0.61±0.03	**0.68±0.01**	0.64±0.01	0.67±0.01
$C218_{EC}$	0.46±0.03	0.35±0.06	0.34±0.03	0.37±0.04	0.48±0.07	0.23±0.05	0.52±0.04	0.56±0.07	0.59±0.02	**0.65±0.01**	0.58±0.04	0.65±0.02
$C219_{Ki}$	0.47±0.02	0.44±0.04	0.4±0.04	0.37±0.04	0.32±0.05	0.46±0.02	0.25±0.03	0.40±0.02	0.32±0.01	**0.48±0.01**	0.31±0.04	0.35±0.03
$C228_{Ki}$	0.64±0.01	0.63±0.02	0.52±0.03	0.58±0.03	0.46±0.11	0.64±0.01	0.64±0.02	0.68±0.02	0.65±0.02	0.68±0.01	0.68±0.01	**0.7±0.01**
$C231_{Ki}$	0.63±0.01	0.58±0.02	0.54±0.03	0.57±0.03	0.64±0.02	0.55±0.0	0.8±0.02	**0.81±0.01**	0.77±0.03	0.79±0.01	0.74±0.0	0.79±0.02
$C233_{Ki}$	0.67±0.0	0.63±0.01	0.59±0.02	0.64±0.02	0.5±0.09	0.7±0.0	0.67±0.02	0.72±0.01	0.69±0.0	0.71±0.01	0.71±0.01	**0.72±0.0**
$C234_{Ki}$	**0.74±0.0**	0.66±0.02	0.6±0.01	0.62±0.04	0.49±0.01	0.71±0.01	0.63±0.01	0.64±0.02	0.67±0.02	0.7±0.01	0.65±0.0	0.69±0.01
$C235_{EC}$	0.53±0.01	0.52±0.02	0.51±0.04	0.48±0.03	0.4±0.1	0.49±0.02	0.66±0.02	0.69±0.02	0.69±0.02	0.72±0.01	0.7±0.02	**0.74±0.01**
$C236_{Ki}$	0.7±0.01	0.7±0.02	0.65±0.02	0.68±0.02	0.52±0.09	0.65±0.02	0.64±0.05	0.74±0.0	0.67±0.01	**0.77±0.01**	0.74±0.01	0.74±0.01
$C237_{EC}$	0.64±0.01	0.6±0.05	0.51±0.03	0.53±0.07	0.52±0.11	0.6±0.0	0.64±0.02	0.62±0.01	0.66±0.02	0.71±0.01	0.66±0.01	**0.72±0.03**
$C237_{Ki}$	0.7±0.0	0.66±0.01	0.67±0.02	0.67±0.02	0.6±0.06	0.67±0.03	0.72±0.01	0.71±0.0	0.68±0.0	**0.75±0.01**	0.71±0.0	0.7±0.01
$C238_{Ki}$	0.65±0.01	0.62±0.04	0.56±0.02	0.63±0.06	0.62±0.01	0.64±0.01	0.63±0.03	0.71±0.0	0.71±0.01	**0.73±0.0**	0.68±0.02	0.7±0.03
$C239_{EC}$	**0.61±0.0**	0.47±0.06	0.41±0.04	0.47±0.07	0.52±0.02	0.58±0.02	0.5±0.01	0.48±0.0	0.44±0.03	0.54±0.02	0.58±0.02	0.58±0.02
$C244_{Ki}$	0.81±0.0	0.8±0.01	0.78±0.01	0.8±0.02	0.63±0.06	0.79±0.0	0.76±0.01	0.8±0.0	0.78±0.01	**0.82±0.0**	0.81±0.0	0.81±0.0
$C262_{Ki}$	0.44±0.04	0.2±0.1	0.34±0.04	0.45±0.02	0.4±0.07	0.58±0.01	0.51±0.03	0.63±0.0	0.6±0.02	**0.65±0.01**	0.6±0.03	0.64±0.03
$C264_{Ki}$	0.65±0.01	0.65±0.03	0.6±0.02	0.62±0.03	0.41±0.04	0.67±0.01	0.62±0.02	0.72±0.0	0.66±0.01	**0.73±0.01**	0.7±0.0	**0.73±0.01**
$C2835_{Ki}$	0.78±0.01	0.78±0.03	0.69±0.04	0.75±0.04	0.7±0.03	0.72±0.01	0.79±0.01	0.79±0.0	0.78±0.02	0.8±0.02	0.76±0.01	**0.82±0.02**
$C287_{Ki}$	0.44±0.01	0.46±0.03	0.21±0.04	0.42±0.05	0.39±0.02	0.46±0.04	0.45±0.02	0.49±0.0	0.34±0.05	**0.57±0.01**	0.5±0.01	0.52±0.0
$C2971_{Ki}$	0.77±0.02	0.72±0.01	0.68±0.02	0.76±0.06	0.78±0.01	0.78±0.01	0.83±0.03	0.81±0.0	0.82±0.01	0.84±0.02	0.85±0.02	**0.85±0.01**
$C3979_{EC}$	**0.66±0.01**	0.36±0.04	0.29±0.03	0.35±0.07	0.34±0.05	0.62±0.05	0.47±0.02	0.26±0.01	0.5±0.01	0.5±0.04	0.48±0.04	0.55±0.05
$C4005_{Ki}$	**0.67±0.01**	0.64±0.03	0.57±0.01	0.44±0.06	0.58±0.07	0.53±0.04	0.57±0.02	0.49±0.01	0.58±0.04	0.63±0.01	0.55±0.02	0.59±0.02
$C4203_{Ki}$	0.12±0.01	0.09±0.12	0.05±0.02	0.09±0.05	0.16±0.03	0.14±0.0	0.16±0.04	0.11±0.0	0.15±0.05	0.33±0.06	0.38±0.04	**0.39±0.03**
$C4616_{EC}$	**0.56±0.01**	0.42±0.06	0.49±0.03	0.48±0.06	0.44±0.1	0.55±0.02	0.43±0.04	0.47±0.0	0.53±0.05	0.52±0.04	0.49±0.02	0.48±0.05
$C4792_{Ki}$	0.59±0.01	**0.65±0.02**	0.55±0.03	0.62±0.04	0.55±0.09	0.64±0.0	0.51±0.03	0.49±0.01	0.49±0.02	0.55±0.01	0.52±0.0	0.54±0.03
Average	0.6235	0.558	0.5253	0.553	0.523	0.5982	0.589	0.6047	0.6025	**0.6614**	0.6279	0.656

Table S3. Fine-tuning performance (R-squared) of MLP, KPGT[19], and Our$_{GPS}$ on 30 binding potency prediction tasks in MoleculeACE[7] using random splits. The performance is averaged across three independent runs, where standard deviation is also shown. Zero standard deviation indicates that the value is below 0.01. Best performance is shown in bold.

**Dataset**	**MLP**	**KPGT[19]**	**Ours$_{GPS}$**
C1862$_{Ki}$	0.7±0.05	0.74±0.03	**0.81±0.0**
C1871$_{Ki}$	0.56±0.06	**0.62±0.03**	0.54±0.03
C2034$_{Ki}$	0.54±0.05	0.54±0.09	**0.65±0.02**
C2047$_{EC}$	**0.57±0.06**	0.34±0.21	0.52±0.04
C204$_{Ki}$	0.75±0.02	0.69±0.01	**0.79±0.01**
C2147$_{Ki}$	0.86±0.01	0.85±0.02	**0.91±0.01**
C214$_{Ki}$	0.63±0.03	0.62±0.02	**0.7±0.01**
C218$_{EC}$	**0.48±0.05**	0.46±0.01	0.43±0.03
C219$_{Ki}$	0.37±0.05	0.47±0.02	**0.54±0.02**
C228$_{Ki}$	0.58±0.04	0.57±0.03	**0.63±0.02**
C231$_{Ki}$	0.68±0.02	0.62±0.1	**0.73±0.02**
C233$_{Ki}$	0.62±0.02	0.6±0.02	**0.72±0.01**
C234$_{Ki}$	0.64±0.03	0.63±0.02	**0.77±0.01**
C235$_{EC}$	0.19±0.21	0.53±0.04	**0.68±0.01**
C236$_{Ki}$	0.65±0.08	0.69±0.02	**0.75±0.01**
C237$_{EC}$	0.63±0.04	0.41±0.09	**0.68±0.02**
C237$_{Ki}$	0.65±0.02	0.65±0.01	**0.76±0.01**
C238$_{Ki}$	0.54±0.07	**0.6±0.02**	0.53±0.03
C239$_{EC}$	0.32±0.12	**0.57±0.01**	0.56±0.01
C244$_{Ki}$	0.75±0.02	0.71±0.0	**0.81±0.01**
C262$_{Ki}$	0.43±0.08	0.44±0.09	**0.55±0.01**
C264$_{Ki}$	0.56±0.04	0.67±0.01	**0.69±0.01**
C2835$_{Ki}$	0.74±0.04	**0.79±0.04**	0.78±0.01
C287$_{Ki}$	0.38±0.11	0.53±0.09	**0.74±0.01**
C2971$_{Ki}$	0.77±0.03	0.74±0.03	**0.85±0.02**
C3979$_{EC}$	0.53±0.05	0.4±0.09	**0.61±0.03**
C4005$_{Ki}$	0.55±0.08	0.56±0.02	**0.59±0.01**
C4203$_{Ki}$	0.27±0.07	**0.3±0.15**	0.24±0.05
C4616$_{EC}$	0.39±0.09	0.31±0.07	**0.48±0.04**
C4792$_{Ki}$	0.54±0.11	0.41±0.16	**0.75±0.0**
Average	0.5625	0.5685	**0.659**

Table S4. Ablation performance (R-squared) from 6 pre-train settings on 30 binding potency prediction tasks in MoleculeACE[7] using stratified splits. This experiment is performed using GIN[11] as model backbone. The performance is averaged across three independent runs, where standard deviation is also shown. Zero standard deviation indicates that the value is below 0.01. Best performance is shown in bold.

Dataset	Full	No regularizations	No intra-channel regularization	No inter-channel regularization	Replace with margin loss	Replace with multi-task learning
$C1862_{Ki}$	**0.79±0.01**	0.74±0.02	0.77±0.02	0.74±0.02	0.77±0.02	0.75±0.01
$C1871_{Ki}$	0.51±0.03	0.47±0.01	0.54±0.04	**0.57±0.01**	0.43±0.02	0.46±0.02
$C2034_{Ki}$	**0.62±0.02**	0.57±0.02	0.6±0.03	0.52±0.02	0.56±0.05	0.58±0.01
$C2047_{EC}$	0.24±0.05	0.35±0.01	0.37±0.06	**0.38±0.09**	0.24±0.04	0.2±0.03
$C204_{Ki}$	**0.8±0.01**	0.79±0.01	**0.8±0.0**	**0.8±0.01**	**0.8±0.0**	0.78±0.01
$C2147_{Ki}$	0.86±0.0	**0.88±0.0**	**0.88±0.01**	0.85±0.0	0.87±0.0	0.87±0.0
$C214_{Ki}$	**0.65±0.01**	0.62±0.01	0.63±0.01	**0.65±0.02**	0.62±0.01	0.64±0.0
$C218_{EC}$	0.48±0.01	0.52±0.01	0.5±0.03	0.5±0.02	**0.59±0.02**	0.5±0.01
$C219_{Ki}$	0.38±0.01	0.32±0.01	0.27±0.03	0.32±0.02	**0.39±0.02**	0.28±0.01
$C228_{Ki}$	0.65±0.01	0.64±0.01	0.61±0.0	0.6±0.01	**0.66±0.0**	0.63±0.0
$C231_{Ki}$	0.75±0.02	0.75±0.02	0.75±0.03	**0.76±0.02**	0.75±0.01	0.75±0.03
$C233_{Ki}$	**0.71±0.01**	0.7±0.01	0.69±0.01	0.69±0.01	0.7±0.01	**0.71±0.01**
$C234_{Ki}$	0.67±0.0	**0.68±0.01**	0.64±0.01	0.65±0.02	**0.68±0.0**	0.66±0.01
$C235_{EC}$	**0.71±0.01**	0.66±0.02	0.67±0.02	0.65±0.01	0.68±0.03	0.66±0.03
$C236_{Ki}$	0.72±0.01	**0.74±0.0**	0.72±0.0	0.71±0.01	0.71±0.01	0.73±0.01
$C237_{EC}$	**0.68±0.02**	0.66±0.02	0.64±0.02	0.66±0.03	0.64±0.02	0.64±0.02
$C237_{Ki}$	0.71±0.0	0.71±0.01	0.69±0.01	0.7±0.01	**0.72±0.01**	**0.72±0.01**
$C238_{Ki}$	0.67±0.01	0.68±0.01	**0.7±0.01**	0.67±0.02	0.63±0.01	0.63±0.04
$C239_{EC}$	0.48±0.01	**0.5±0.01**	0.48±0.03	0.45±0.02	0.45±0.01	0.48±0.02
$C244_{Ki}$	0.81±0.0	0.76±0.01	0.78±0.01	0.79±0.01	**0.82±0.0**	0.81±0.01
$C262_{Ki}$	0.63±0.02	**0.65±0.02**	0.57±0.02	**0.65±0.0**	0.59±0.02	0.52±0.02
$C264_{Ki}$	0.69±0.01	0.69±0.02	0.68±0.01	**0.7±0.01**	0.69±0.02	0.66±0.01
$C2835_{Ki}$	0.8±0.01	**0.82±0.0**	0.78±0.01	0.77±0.01	**0.82±0.01**	0.78±0.02
$C287_{Ki}$	0.53±0.01	0.55±0.0	0.5±0.02	0.51±0.02	0.53±0.01	**0.56±0.01**
$C2971_{Ki}$	0.83±0.01	0.83±0.0	0.8±0.01	**0.85±0.01**	**0.85±0.01**	0.81±0.0
$C3979_{EC}$	0.52±0.02	0.45±0.02	0.5±0.01	**0.55±0.02**	0.52±0.02	0.52±0.03
$C4005_{Ki}$	**0.59±0.01**	0.58±0.01	0.56±0.03	0.56±0.01	**0.59±0.0**	0.55±0.02
$C4203_{Ki}$	**0.32±0.02**	0.26±0.04	0.28±0.05	0.28±0.01	0.24±0.03	0.21±0.03
$C4616_{EC}$	**0.49±0.03**	0.38±0.02	0.44±0.01	0.4±0.01	0.44±0.02	**0.49±0.04**
$C4792_{Ki}$	0.55±0.01	0.53±0.01	0.48±0.04	0.55±0.03	0.55±0.01	**0.56±0.01**
Average	**0.6288**	0.6169	0.6112	0.6162	0.6173	0.6045

Table S5. Ablation performance (R-squared) of 7 fine-tune strategies on 30 binding potency prediction tasks in MoleculeACE[7] using stratified splits. This experiment is performed using GIN[11] as model backbone. The performance is averaged across three independent runs, where standard deviation is also shown. Zero standard deviation indicates that the value is below 0.01. Best performance is shown in bold.

Dataset	PW *t=1*	Sparse PW *t=0.7*	Sparse PW *t=0.3*	Concat	MoE *t=1*	Sparse MoE *t=0.7*	Sparse MoE *t=0.3*
$C1862_{Ki}$	**0.772±0.01**	0.77±0.01	0.760±0.01	0.771±0.0	0.767±0.01	0.751±0.02	0.708±0.03
$C1871_{Ki}$	0.505±0.01	0.54±0.01	**0.549±0.01**	0.539±0.01	0.532±0.03	0.509±0.03	0.454±0.04
$C2034_{Ki}$	0.492±0.01	0.52±0.02	0.523±0.04	0.508±0.02	0.530±0.03	**0.533±0.02**	0.475±0.02
$C2047_{EC}$	0.245±0.05	**0.32±0.05**	0.204±0.01	0.209±0.03	0.239±0.08	0.253±0.02	0.232±0.04
$C204_{Ki}$	0.796±0.01	0.79±0.01	**0.798±0.01**	0.786±0.0	0.786±0.01	0.757±0.01	0.776±0.02
$C2147_{Ki}$	**0.876±0.01**	0.87±0.0	0.873±0.01	0.856±0.01	0.861±0.01	0.865±0.01	0.851±0.01
$C214_{Ki}$	**0.654±0.01**	0.64±0.01	0.615±0.01	0.630±0.03	0.634±0.03	0.643±0.02	0.627±0.01
$C218_{Ki}$	0.582±0.02	0.58±0.04	**0.585±0.02**	0.581±0.01	0.573±0.0	0.570±0.02	0.583±0.01
$C219_{Ki}$	**0.398±0.01**	0.31±0.04	0.383±0.02	0.349±0.01	0.358±0.02	0.331±0.03	0.332±0.01
$C228_{Ki}$	**0.694±0.01**	0.68±0.01	0.648±0.0	0.692±0.02	0.657±0.02	0.662±0.05	0.661±0.03
$C231_{Ki}$	0.729±0.01	0.74±0.0	0.734±0.01	**0.751±0.01**	0.733±0.02	0.723±0.02	0.715±0.02
$C233_{Ki}$	0.708±0.01	**0.71±0.01**	0.672±0.02	0.707±0.01	0.701±0.02	0.687±0.02	0.652±0.01
$C234_{Ki}$	0.669±0.01	0.65±0.0	0.668±0.01	0.682±0.02	0.656±0.02	**0.684±0.02**	0.650±0.02
$C235_{EC}$	0.665±0.01	**0.7±0.02**	0.651±0.01	0.690±0.01	0.675±0.01	0.694±0.0	0.699±0.02
$C236_{Ki}$	**0.750±0.0**	0.74±0.01	0.741±0.01	0.731±0.0	0.707±0.01	0.711±0.01	0.707±0.01
$C237_{EC}$	**0.671±0.03**	0.66±0.01	0.658±0.0	0.669±0.01	0.651±0.02	0.627±0.01	0.648±0.03
$C237_{Ki}$	**0.724±0.0**	0.71±0.0	0.716±0.0	0.706±0.01	0.695±0.01	0.707±0.02	0.708±0.01
$C238_{Ki}$	0.681±0.01	0.68±0.2	0.692±0.01	**0.703±0.01**	0.682±0.01	0.658±0.02	0.625±0.03
$C239_{EC}$	0.445±0.02	**0.58±0.02**	0.436±0.01	0.465±0.01	0.472±0.04	0.496±0.02	0.511±0.03
$C244_{Ki}$	0.809±0.01	**0.81±0.0**	0.807±0.0	0.802±0.0	0.794±0.01	0.786±0.01	0.781±0.01
$C262_{Ki}$	0.594±0.01	0.6±0.03	**0.610±0.02**	**0.610±0.01**	0.515±0.07	0.584±0.01	0.577±0.07
$C264_{Ki}$	0.681±0.01	**0.7±0.0**	0.688±0.01	0.661±0.01	0.692±0.01	0.685±0.01	0.645±0.02
$C2835_{Ki}$	0.795±0.01	0.76±0.01	0.762±0.01	0.789±0.0	0.777±0.01	**0.797±0.0**	0.791±0.0
$C287_{Ki}$	0.509±0.01	0.5±0.01	0.493±0.0	0.471±0.02	**0.523±0.02**	0.508±0.02	0.455±0.02
$C2971_{Ki}$	0.848±0.02	0.85±0.02	0.820±0.01	**0.861±0.0**	0.820±0.02	0.792±0.02	0.792±0.02
$C3979_{EC}$	0.500±0.06	0.48±0.04	**0.544±0.03**	0.537±0.02	0.526±0.05	0.539±0.02	0.490±0.03
$C4005_{Ki}$	0.583±0.01	0.55±0.02	**0.608±0.01**	0.601±0.03	0.548±0.03	0.541±0.0	0.558±0.02
$C4203_{Ki}$	**0.384±0.02**	0.38±0.04	0.335±0.04	0.293±0.04	0.292±0.06	0.300±0.01	0.284±0.05
$C4616_{EC}$	0.458±0.04	0.49±0.02	**0.529±0.06**	0.467±0.02	0.503±0.03	0.470±0.03	0.452±0.04
$C4792_{Ki}$	0.533±0.01	0.52±0.0	0.544±0.02	**0.548±0.01**	0.486±0.04	0.537±0.01	0.521±0.03
Average	0.6250	**0.6279**	0.6215	0.6222	0.6128	0.6133	0.5987

References

1. Sterling, T. & Irwin, J. J. Zinc 15 – ligand discovery for everyone. *J. Chem. Inf. Model.* **55**, 2324–2337 (2015).
2. Morgan, H. L. The generation of a unique machine description for chemical structures-a technique developed at chemical abstracts service. *J. Chem. Documentation* **5**, 107–113 (1965).
3. Rong, Y. *et al.* Self-supervised graph transformer on large-scale molecular data. In *Proc. NeurIPS 2020* (2020).
4. Aldeghi, M. *et al.* Roughness of Molecular Property Landscapes and Its Impact on Modellability. *J. Chem. Inf. Model.* **62**, 4660–4671, (2022).
5. Zhang, Z., Bian, Y., Xie, A., Han, P. & Zhou, S. Can pretrained models really learn better molecular representations for ai-aided drug discovery? *J. Chem. Inf. Model.* **64**, 2921–2930 (2024).
6. Wu, Z. *et al.* Moleculenet: a benchmark for molecular machine learning. *Chem. Sci.* **9**, 513–530 (2018).
7. van Tilborg, D., Alenicheva, A. & Grisoni, F. Exposing the limitations of molecular machine learning with activity cliffs. *J. Chem. Inf. Model.* **62**, 5938–5951, (2022).
8. Xu, M., Wang, H., Ni, B., Guo, H. & Tang, J. Self-supervised graph-level representation learning with local and global structure. *PMLR*, **139**, 11548–11558 (2021).
9. Wang, Y., Wang, J., Cao, Z. & Barati Farimani, A. Molecular contrastive learning of representations via graph neural networks. *Nat. Mach. Intell.* **4**, 279–287, (2022).
10. Hubert, L. & Arabie, P. Comparing partitions. *J. Classif.* **2**, 193–218, (1985).
11. Xu, K., Hu, W., Leskovec, J. & Jegelka, S. How powerful are graph neural networks? In *Proc. ICLR 2019* (2019).
12. Shazeer, N. *et al.* Outrageously large neural networks: The sparsely-gated mixture-of-experts layer. In *Proc. ICLR 2017* (2017).
13. Zhang, S., Liu, Y. & Xie, L. A universal framework for accurate and efficient geometric deep learning of molecular systems. *Sci. Reports* **13**, 19171, (2023).
14. Zhou, G. *et al.* Uni-mol: A universal 3d molecular representation learning framework. In *Proc. ICLR 2023* (2023).
15. Fang, X. *et al.* Geometry-enhanced molecular representation learning for property prediction. *Nat. Mach. Intell.* **4**, 127–134, (2022).
16. Liu, S. *et al.* Pre-training molecular graph representation with 3d geometry. In *Proc. ICLR 2022* (2022).
17. Ross, J. *et al.* Large-scale chemical language representations capture molecular structure and properties. *Nat. Mach. Intell.* **4**, 1256–1264, (2022).
18. Fang, Y. *et al.* Knowledge graph-enhanced molecular contrastive learning with functional prompt. *Nat. Mach. Intell.* **5**, 542–553 (2023).
19. Li, H. *et al.* A knowledge-guided pre-training framework for improving molecular representation learning. *Nat. Commun.* **14**, 7568, (2023).
